# Protective Effect of Intracellular Compounds of *Lactobacillus plantarum* on Goat Sperm Quality During Cryopreservation

**DOI:** 10.1002/vms3.70850

**Published:** 2026-04-15

**Authors:** Farshad Ariyan, Amjad Farzinpour, Abbas Farshad, Aram Sharifi

**Affiliations:** ^1^ Department of Animal Science Faculty of Agriculture University of Kurdistan Sanandaj Iran

**Keywords:** antioxidants, extender, freezing‐thawing, intracellular metabolome

## Abstract

**Background:**

Cryopreservation of sperm is a key strategy for preserving and transmitting the genetic traits of valuable or endangered breeds and is particularly applied in goats to improve sperm quality and performance.

**Objectives:**

This study was conducted to evaluate the effect of supplementing cryopreservation extenders with the intracellular compounds of *Lactobacillus plantarum* (ICL) on goat sperm quality.

**Methods:**

The intracellular compounds were added to the semen extender at concentrations of 20, 40, 60, 80 and 100 µL/mL, while the control group received only the basic extender without any supplementation. Following freezing and thawing, sperm quality parameters were assessed.

**Results:**

Among the 10 intracellular compounds identified in *L. plantarum*, the highest proportions corresponded to 1‐benzenesulfonyl‐1H‐pyrrole and benzenesulfonyl azide (CAS). Treatment with ICL20 significantly increased total motility compared with the control group (*p* < 0.05). Both ICL20 and ICL40 significantly improved progressive motility compared with the control and ICL100 groups. The mean amplitude of lateral head displacement (ALH, µm) was significantly higher in ICL20 and ICL40 compared with the control and ICL100. Moreover, ICL20 showed significantly higher beat/cross frequency (BCF, Hz) than the control, ICL80 and ICL100 groups. Treatments ICL20 and ICL40 also exhibited significantly greater viability compared with the control and ICL100. Malondialdehyde (MDA) concentration was significantly reduced in ICL20 and ICL40 compared with the control, ICL80 and ICL100. Sperm chromatin dispersion (SCD) analysis revealed that ICL20 had a significantly higher halo‐to‐core ratio compared with all other treatments.

**Conclusion:**

Overall, the findings indicate that supplementation of goat semen extenders with ICL, particularly at concentrations of 20 and 40 µL/mL, improves sperm quality during cryopreservation and thawing.

## Graphical abstract

Intracellular compounds from *Lactobacillus plantarum* were added to a goat sperm cryopreservation extender. A concentration of 20 µL/mL improved post‐thaw sperm quality, whereas higher concentrations (80–100 µL/mL) had detrimental effects.

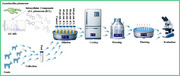

## 1 Introduction

Sperm cryopreservation is a practical technique for promoting breeding programs and conserving valuable or endangered breeds. Despite significant advances in sperm cryopreservation and artificial insemination in cattle, the use of frozen semen and artificial insemination in goats remains challenging due to the reduced quality of thawed sperm (Longobardi et al. 2020). During cryopreservation, sperm are exposed to cold shock, lipid peroxidation (LPO), and increased production of reactive oxygen species (ROS), all of which contribute to the decline in sperm quality (Condorelli et al. 2017; Santiago‐Moreno et al. 2017; B. Zhang et al. 2021). This high sensitivity of mammalian sperm is primarily attributed to the elevated concentration of unsaturated fatty acids in the sperm membrane, making them more susceptible to LPO in the presence of ROS and less resistant to cold shock (Van Tran et al. 2016; Craig et al. 2019).

ROS are essential by‐products generated by cells and are normally maintained in balance with the body's antioxidant system (Malo et al. 2011; G. Zhang et al. 2019). However, oxidative stress can arise when this balance is disrupted (Foutouhi and Meyers 2022; Mateo‐Otero et al. 2024; Dash et al. 2025). While sperm cells and seminal plasma possess intrinsic antioxidant systems to counteract ROS, the unique morphology of sperm limits antioxidant levels, potentially leading to elevated ROS and disruption of cellular homeostasis (Shrivastava et al. 2021; Wang et al. 2025). As a result, ROS can induce oxidative stress by reacting with lipids, proteins and DNA, which may reduce sperm motility, decrease viability, compromise membrane integrity, increase DNA damage and elevate MDA levels (Nickavar et al. 2010; Fujii and Imai 2014; Bahmyari et al. 2020; Mustofa et al. 2021).

Given these challenges, the composition of the extender used during cryopreservation plays a crucial role in maintaining sperm integrity (Alcay et al. 2016; Toker et al. 2016; Bodu et al. 2025). Consequently, supplementation of semen extenders with antioxidants and cryoprotective compounds to reduce ROS levels and freezing‐induced damage has attracted considerable attention. The beneficial effects of adding antioxidants and cryoprotective compounds to sperm extenders have been demonstrated in numerous species, including goats (Dalmazzo et al. 2018; Ariyan et al. 2021; Bang et al. 2021; Azimi et al. 2020; Rezaei et al. 2023; Abdelnour et al. 2023; Ali et al. 2024; Kumar et al. 2024; Yi et al. 2024; Ahmet et al. 2025; Ariyan et al. 2025; Shahid et al. 2025).

Among potential antioxidant and protective sources, *Lactobacillus plantarum* has been extensively studied (Sentürk et al. 2020). *L. plantarum* species are important for regulating the human gut microbiota and maintaining homeostasis (Song et al. 2000). Lactic acid bacteria (LAB), including *L. plantarum*, contribute to human health by producing various cellular structures and metabolites, such as cell surface components, short‐chain fatty acids (SCFAs) and bioactive peptides (Salminen et al. 2021). Notably, according to these reports (J. Lee et al. 2005; Shen et al. 2011; Aguilar‐Toalá et al. 2019), ICL exhibit antioxidant and membrane‐protective properties and may neutralize peroxyl and lipophilic radicals generated during membrane LPO, protecting cellular structures from oxidative damage. This indicates that ICL may contain lipophilic antioxidants that help prevent membrane damage and protect biological systems from oxidative stress via hydrogen atom or single‐electron transfer to proteins and lipids (Aguilar‐Toalá et al. 2019).

Furthermore, previous studies have highlighted the biological potential of *L. plantarum*. J. Y. Kim et al. (2002) reported that daily administration of 100 and 200 mg/kg body weight of ICL exhibited antitumour activity in tumour‐bearing mice. Moreover, Aguilar‐Toalá et al. (2019) demonstrated that ICL can mitigate oxidative stress in red blood cells, exert protective effects on the erythrocyte membrane and reduce oxidative haemolysis by decreasing membrane deformability. In addition, Dim et al. (2020) reported that supplementation of *L. plantarum* in drinking water improved semen quality in local turkeys, enhancing semen volume, sperm concentration, progressive motility, total motility and the percentage of live and morphologically normal sperm compared to controls.

Despite extensive research on the antioxidant and protective properties of ICL, no studies have directly evaluated its effects on sperm during cryopreservation. Evidence demonstrating the antioxidant and membrane‐protective activity of ICL (J. Lee et al. 2005; Shen et al. 2011; Aguilar‐Toalá et al. 2019) suggests that supplementation of sperm extenders with ICL may help mitigate oxidative stress and cryopreservation‐induced damage. Therefore, the present study was conducted to evaluate the protective effects of ICL on goat semen quality during freezing and thawing.

## 2 Materials and Methods

All experimental procedures in this study were conducted in accordance with international guidelines and were approved by the Animal Care and Use Committee of the University of Kurdistan, Sanandaj, Kurdistan, Iran (Ethical Approval Code: IR.UOK.REC.1404.021).

### 2.1 Chemicals

All chemical reagents used in this study were purchased from Sigma‐Aldrich (St. Louis, MO, USA) and Merck (Darmstadt, Germany).

### 2.2 Extraction of ICL

ICL were extracted using a standardized cold methanol extraction protocol adapted from previously reported methods with fixed parameters to ensure reproducibility. Briefly, *L. plantarum* cultures were grown to the mid‐log phase and immediately placed on ice to halt metabolic activity. For each biological replicate, 1 mL of culture was transferred into a 2 mL microtube and centrifuged at 15,000 × *g* for 10 min at 4°C to pellet the cells. The supernatant was discarded, and the pellet was washed three times with ice‐cold PBS (pH 7.4). Each washing step was followed by centrifugation at 5000 × *g* for 10 min at 4°C.

After washing, 250 µL of cold methanol (−20°C) was added to each pellet, and the suspension was vortexed vigorously for 1 min. The samples were then incubated at −20°C for 20 min to facilitate ICL extraction. To improve cell disruption, samples were sonicated on ice at 200 W with 2 s on/3 s off pulses for a total duration of 4 min. Following extraction, samples were centrifuged at 15,000 × *g* for 15 min at 4°C, and the clarified supernatants were transferred to pre‐chilled tubes.

Approximately, 150 µL of extract from each sample was dried in a vacuum concentrator. The dried residue was reconstituted in 50% acetonitrile and 50% ultrapure water (v/v), vortexed thoroughly and stored at 4°C until GC‐MS analysis. For long‐term storage, aliquots were kept at −80°C.

All buffers, solvents and consumables were pre‐chilled, and extractions were performed on ice to minimize enzymatic activity and maintain metabolite stability (Schelli et al. 2017; Xu et al. 2017; Yang et al. 2018; S. H. Kim et al. 2023).

### 2.3 Analysis of ICL

Gas chromatography–mass spectrometry (GC‐MS) was employed to characterize ICL (Ali et al. 2024).

### 2.4 Preparation of Spermatozoa

This study was conducted at the University of Kurdistan farm, located in Sanandaj, Iran. For this experiment, semen samples were collected from four mature Markhoz bucks (3–4 years old, weighing 60–70 kg) via electro‐ejaculation, twice a week. The samples were promptly transported to the laboratory within 10–15 min after collection. Upon arrival, the semen samples were maintained in a water bath at 37°C and their initial quality was assessed. Samples exhibiting progressive motility greater than 75% and sperm concentration exceeding 1 × 10^9^/mL were selected for subsequent experiments. In addition, at each stage, semen from the four bucks was pooled to minimize individual variation. All experiments were performed in eight replicates.

In this study, a basic extender was used, consisting of 3.786 g Tris, 2.172 g citric acid and 1 g fructose per 100 mL of distilled water. The basic extender was supplemented with 5% (v/v) glycerol and 5% (v/v) egg yolk. According to the method described by Evans and Maxwell (1987), the extender was adjusted to an osmolality of 320 mOsm/kg and a pH of 6.8.

The experimental treatments included a basic extender without any additive (serving as the control group) and the basic extender supplemented with 20, 40, 60, 80 and 100 µL/mL of ICL. Semen was diluted to a final concentration of 240 × 10^6^ spermatozoa/mL with the respective extenders, and aliquots were then placed in 0.25 mL straws. After sealing the straws with polyvinyl chloride powder, they were subjected to a cooling phase at 4°C for 3 h.

During the freezing phase, the straws were placed 4 cm above the surface of liquid nitrogen for 15 min and subsequently immersed in liquid nitrogen at −196°C before being stored in nitrogen tanks. For the thawing procedure, the straws were placed in a 37°C water bath for 30 s and then prepared for subsequent evaluations.

### 2.5 Assessment of Sperm Motion Characteristics

Sperm motility and kinematic parameters were assessed using a computer‐assisted sperm analysis system (CASA: IVOS version 12; Hamilton‐Thorne Biosciences, MA, USA). Five microlitres of diluted semen were placed on a pre‐warmed chamber slide (Leja 4, Leja Products, Luzernestraat B.V., Holland), and sperm motility characteristics were evaluated under a 10× objective lens at 37°C. The motility parameters analysed included total motility (%), progressive motility (%), average path velocity (VAP, µm/s), straight‐line velocity (VSL, µm/s), curvilinear velocity (VCL, µm/s), ALH, BCF, linearity (LIN % = VSL/VCL) and straightness (STR % = VSL/VAP). For each evaluation, 10 microscopic fields were analysed to include a minimum of 200 sperm cells per sample (Bucak et al. 2010).

### 2.6 Sperm Viability

Sperm viability was evaluated using the eosin–nigrosin staining method. Five microlitres of thawed semen were placed on a microscope slide and mixed with 10 µL of eosin–nigrosin solution. The stained sperm were then spread evenly on the slide. A total of 200 spermatozoa were examined under a light microscope at 40× magnification, and the numbers of live (unstained) and dead (stained) sperm were recorded (Evans and Maxwell 1987).

### 2.7 Sperm Membrane Integrity

The functional integrity of the sperm plasma membrane was assessed using the hypo‐osmotic swelling (HOS) test (Revell and Mrode 1994).

The hypo‐osmotic solution consisted of 9 g fructose and 4.9 g sodium citrate dissolved in 1 L of distilled water, with an osmotic pressure of 100 mOsm. In this environment, the tails of sperm with intact membranes swell and curl. Ten millilitres of thawed semen were incubated with 100 mL of the hypo‐osmotic solution at 37°C for 60 min. After incubation, a drop of the mixture was placed on a pre‐warmed slide and covered with a cover slip. A total of 200 sperm cells were examined across at least five different microscopic fields at 400× magnification, and the percentage of sperm with swollen and coiled tails, indicative of intact plasma membranes, was calculated.

### 2.8 Sperm Acrosomal Integrity

For the assessment of acrosome integrity, a formalin–citrate buffer solution was prepared, consisting of 96 mL of 2.9% sodium citrate solution and 4 mL of 37% formaldehyde. Thawed semen samples were fixed in this solution at a ratio of 1:2. A drop of the fixed sample was placed on a microscope slide and covered with a cover slip. A total of 200 spermatozoa per slide were examined under a light microscope (Nikon, Tokyo, Japan) at 1000× magnification, and the percentage of sperm with intact acrosomes was recorded as viable sperm (Weitz 1977).

### 2.9 LPO

LPO in semen samples was assessed using the thiobarbituric acid (TBA) assay to measure MDA concentrations. Semen samples were first thawed and centrifuged at 1500 × *g* for 5 min to separate the supernatant. Then, 1 mL of the supernatant was mixed with 1 mL of EDTA solution (0.037 g EDTA in 10 mL distilled water), 1 mL of BHT solution (0.2 g BHT in 10 mL ethanol) and 2 mL of TCA solution (3 g TCA in 30 mL distilled water). The mixture was centrifuged at 1200 × *g* for 15 min. Subsequently, 1 mL of the resulting supernatant was combined with 1 mL of TBA solution (0.134 g TBA in 20 mL distilled water) and incubated in a water bath at 90°C for 20 min. After cooling to room temperature, absorbance was measured at 532 nm using a spectrophotometer, and MDA concentrations were expressed in nmol/mL (Esterbauer and Cheeseman 1990).

### 2.10 SCD Test

Sperm DNA fragmentation in frozen‐thawed samples was assessed using a modified protocol (Fernández et al. 2003).

Initially, 150 µL of 0.65% agarose was placed on a microscope slide, covered with a coverslip and kept at 4°C for 5 min to form a solid agarose layer. The coverslip was then removed. Next, 30 µL of semen sample was mixed with 70 µL of 0.7% low‐melting‐point agarose and layered onto the agarose‐coated slide. The slide was again covered with a coverslip and allowed to set at room temperature. After removal of the coverslip, the slide was horizontally incubated in the dark at 37°C in an acidic denaturation solution (0.08 N HCl) for 7 min. The slide was then transferred to a lysis solution (0.4 M Tris base, 0.8 M DTT, 1% SDS, 50 mM EDTA and 2 M NaCl, pH 7.5) for 25 min. Following lysis, the slide was washed with distilled water for 5 min and sequentially dehydrated in 70%, 90% and 100% ethanol for 2 min each. After air‐drying at room temperature, the slide was stained with ethidium bromide. Sperm were evaluated using a fluorescence microscope, and the areas of the halo and sperm head nuclei were measured. The ratio of halo area to nuclear area was calculated to assess DNA damage among different treatments (Figure 2).

### 2.11 Statistical Analysis

Data were analysed using the PROC GLM procedure of SAS (version 9.1; SAS Institute, 2002, Cary, NC, USA) in a completely randomized design. Orthogonal contrasts were used to compare means, and statistical significance was set at *p* < 0.05. Results are presented as mean ± standard error (SE).

## 3 Results

**FIGURE 1:**
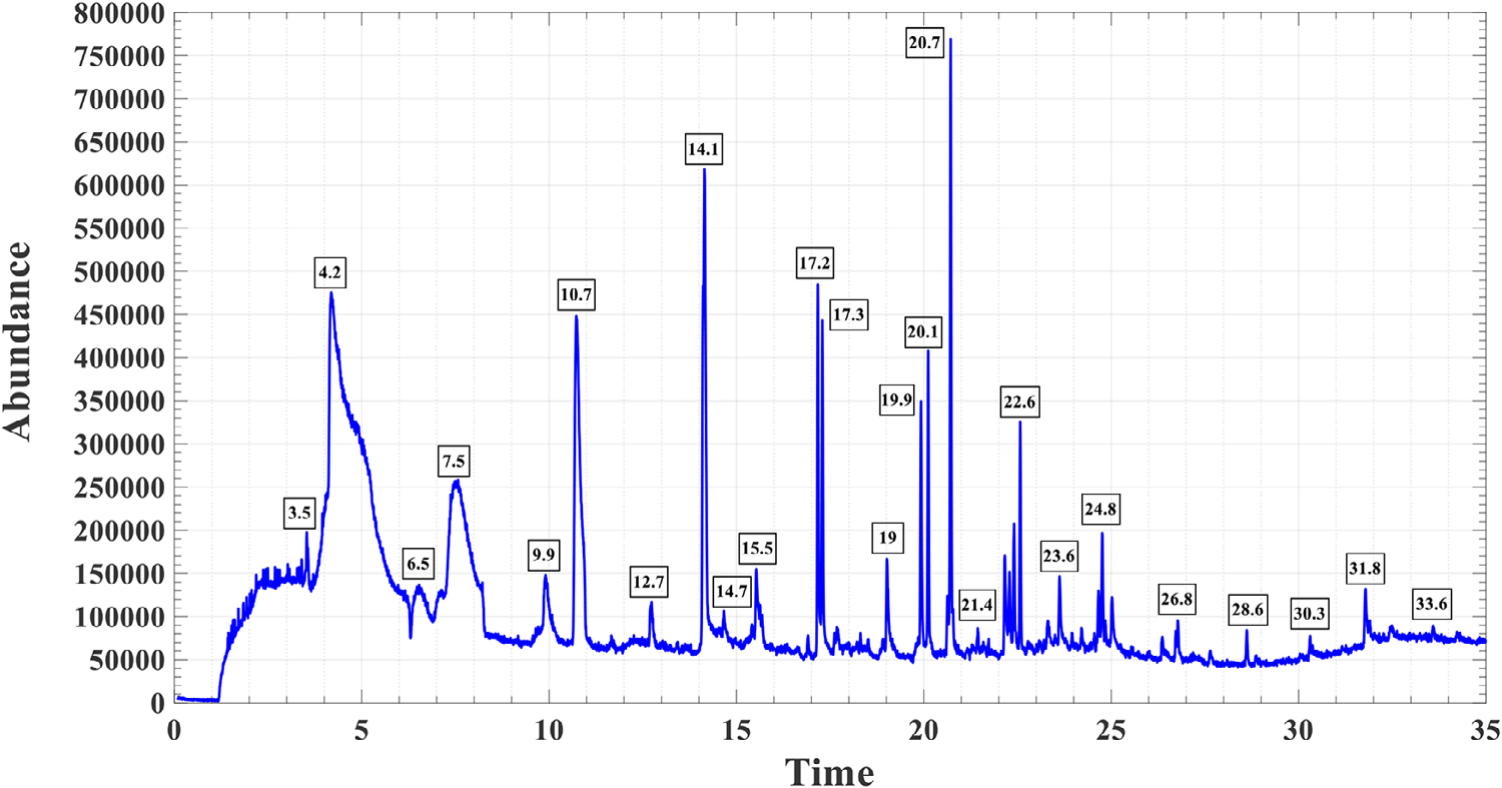
GC‐MS spectrum of intracellular compounds extracted from *Lactobacillus plantarum* (GC‐MS: Gas Chromatography‐Mass Spectrometry).

The ICL were identified using GC–MS. A total of 10 compounds were detected, with the highest proportions corresponding to 1‐benzenesulfonyl‐1H‐pyrrole and benzenesulfonyl azide (CAS) (Table 1 and Figure 1 ).

**TABLE 1:** Intracellular compounds of *Lactobacillus plantarum* (ICL) identified by GC–MS.

Compound number	RT (mint)	Name	% of total
1	3.033	Benzenesulfonyl azide (CAS)	23.95
2	3.403	2‐Propanone, 1‐(ethylthio)‐ (CAS)	1.09
3	3.541	(Ethylthio)‐2‐propanone	3.30
4	4.194	1‐Benzenesulfonyl‐1H‐pyrrole	59.00
5	6.562	Oxime‐, methoxy‐phenyl‐_	4.10
6	9.912	4‐Ethyl‐3‐methyl‐9H‐carbazole‐2‐carboxylate	2.04
7	19.926	1,1,3,3,5,5,7,7,9,9,11,11,13,13,15,15‐HEXADECAMETHYL‐OCTASILOXANE	1.64
8	20.717	Cyclohexane, 1‐ethyl‐1‐methyl‐ (CAS)	3.63
9	22.167	Octadecane	0.68
10	22.288	Hexadecane, 2,6,10,14‐tetramethyl‐	0.58

**FIGURE 2:**
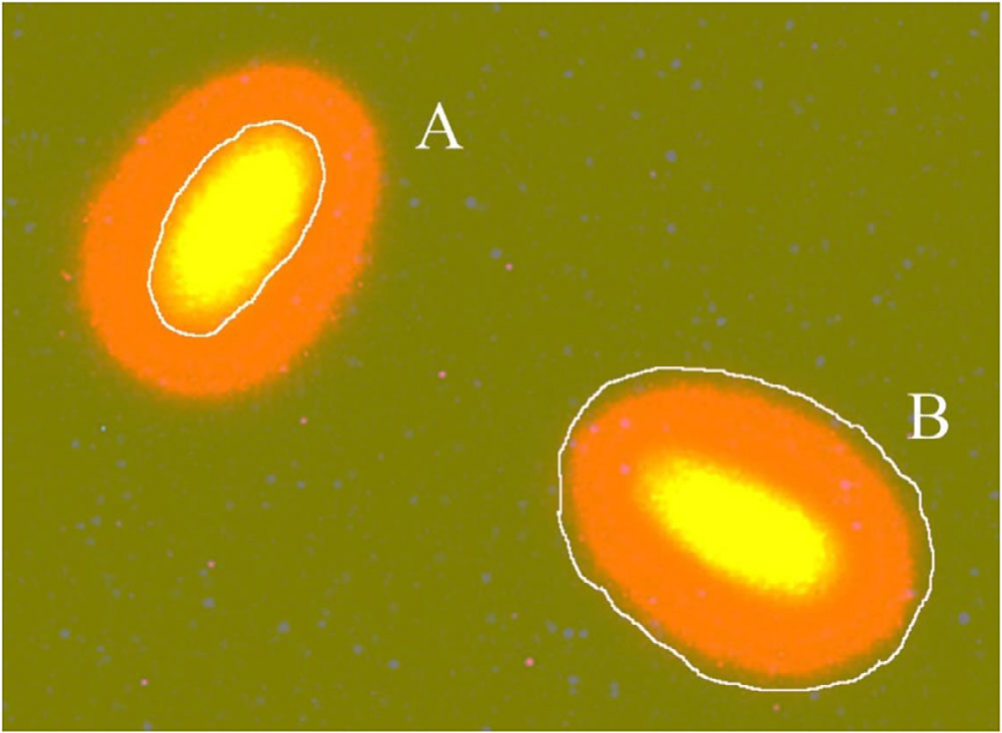
Fluorescence image of sperm nucleoid stained with ethidium bromide showing a central core (A) and a surrounding halo of dispersed DNA (B). DNA integrity is evaluated by the ratio of halo area to core area (Fernández et al. 2003).

Data presented in Table 2 indicate that treatment with ICL20 significantly improved total motility compared to the control and ICL100 treatments (*p* < 0.05). In addition, total motility in the ICL40 group was significantly higher than in the ICL100 group. Both ICL20 and ICL40 treatments significantly increased progressive motility compared to the control and ICL100 groups. Regarding VSL, the ICL20 treatment exhibited significantly higher values compared to ICL100. The mean ALH in the ICL20 group was significantly higher than in the control, ICL60, ICL80 and ICL100 groups; furthermore, ALH in ICL40 was significantly higher than in the control and ICL100 groups. ICL20 treatment also resulted in a significant increase in LIN compared to ICL100. BCF was significantly enhanced in ICL20 relative to the control, ICL80 and ICL100 groups, while ICL40 significantly increased BCF compared to ICL80 and ICL100. No significant differences were observed among the treatments for VAP, VCL and STR.

**TABLE 2:** Effect of different concentrations of intracellular compounds of *Lactobacillus plantarum* (ICL) on sperm motility parameters of goat after thawing (mean ± SE).

Parameters	Control	ICL20	ICL40	ICL60	ICL80	ICL100
Total motility (%)	51.38 ± 1.19^bc^	57.00 ± 1.34^a^	56.38 ± 1.40^ab^	54.00 ± 1.30^abc^	52.38 ± 2.31^abc^	50.38 ± 2.89^c^
Progressive motility (%)	35.63 ± 2.73^b^	44.00 ± 2.66^a^	42.50 ± 1.79^a^	40.00 ± 2.29^ab^	38.00 ± 1.77^ab^	34.00 ± 1.87^b^
VAP (µm/s)	51.48 ± 3.33	55.59 ± 2.93	54.89 ± 2.94	52.00 ± 1.77	51.33 ± 2.01	48.39 ± 1.94
VSL (µm/s)	33.69 ± 1.37^ab^	37.57 ± 2.29^a^	35.79 ± 2.65^ab^	34.17 ± 1.57^ab^	33.48 ± 1.27^ab^	30.30 ± 2.38^b^
VCL (µm/s)	131.17 ± 3.51	134.69 ± 3.56	132.87 ± 4.27	130.53 ± 2.29	130.38 ± 3.23	127.15 ± 4.34
ALH (µm)	5.98 ± 0.11^cd^	7.15 ± 0.24^a^	6.80 ± 0.29^ab^	6.37 ± 0.16^bc^	6.34 ± 0.20^bc^	5.72 ± 0.16^d^
STR (%)	67.64 ± 5.59	68.36 ± 4.19	65.44 ± 4.33	65.63 ± 1.31	65.30 ± 0.91	63.69 ± 6.62
LIN (%)	25.79 ± 1.16^ab^	28.05 ± 1.89^a^	26.86 ± 1.73^ab^	26.13 ± 0.86^ab^	25.76 ± 1.01^ab^	23.82 ± 1.57^b^
BCF (Hz)	11.93 ± 0.79^bc^	13.74 ± 0.51^a^	13.59 ± 0.82^ab^	12.78 ± 0.49^abc^	11.82 ± 0.62^c^	11.37 ± 0.29^c^

*Note*: Linearity (LIN %) = VSL/VCL; Straightness (STR %) = VSL/VAP.

Abbreviations: ALH, amplitude of lateral head displacement; BCF, beat/cross frequency; ICL, intracellular compounds of *Lactobacillus plantarum*; VAP, average path velocity; VCL, curvilinear velocity; VSL, straight‐line velocity.

^a, b, c^Different superscripts within the same row demonstrate significant differences (*p* < 0.05).

Results of membrane integrity, sperm viability and acrosomal integrity are presented in Table 3. Treatments ICL20 and ICL40 significantly improved membrane integrity compared to ICL80 and ICL100. Regarding sperm viability, ICL20 showed significantly higher viability than the control, ICL60, ICL80 and ICL100 groups, while ICL40 also exhibited significantly higher viability than the control and ICL100. None of the experimental treatments showed a significant difference in the acrosomal integrity parameter compared to each other.

**TABLE 3:** Effects of different concentrations of intracellular compounds of *Lactobacillus plantarum* (ICL) on qualitative sperm parameters of goat after thawing (mean ± SE).

Parameters	Control	ICL20	ICL40	ICL60	ICL80	ICL100
Membrane integrity (%)	51.94 ± 1.34^ab^	55.75 ± 1.78^a^	55.69 ± 1.58^a^	54.50 ± 1.40^ab^	50.94 ± 1.68^b^	50.56 ± 1.68^b^
Acrosome statue (%)	65.63 ± 2.03	66.38 ± 1.64	64.94 ± 1.13	64.81 ± 1.31	64.81 ± 1.20	64.69 ± 2.05
Viability (%)	59.25 ± 1.64^c^	66.69 ± 1.49^a^	64.00 ± 1.55^ab^	61.19 ± 1.39^bc^	61.00 ± 1.56^bc^	58.13 ± 1.95^c^
SCD stained HS/WNS (%)	2.57 ± 0.14^bc^	3.94 ± 0.36^a^	3.05 ± 0.08^b^	2.80 ± 0.11^b^	2.56 ± 0.16^bc^	2.14 ± 0.09^c^
Malondialdehyde concentration (nmol/mL)	3.13 ± 0.29^c^	2.10 ± 0.14^a^	2.42 ± 0.20^ab^	2.90 ± 0.16^bc^	3.15 ± 0.17^c^	3.41 ± 0.08^c^

Abbreviation: ICL, intracellular compounds of *Lactobacillus plantarum*.

^a, b, c^Different superscripts within the same row demonstrates significant differences (*p* < 0.05).

MDA concentrations, shown in Table 3, were significantly reduced in ICL20 compared to the control, ICL60, ICL80 and ICL100 groups; ICL40 similarly showed a significant decrease compared to the control, ICL80 and ICL100.

**FIGURE 3:**
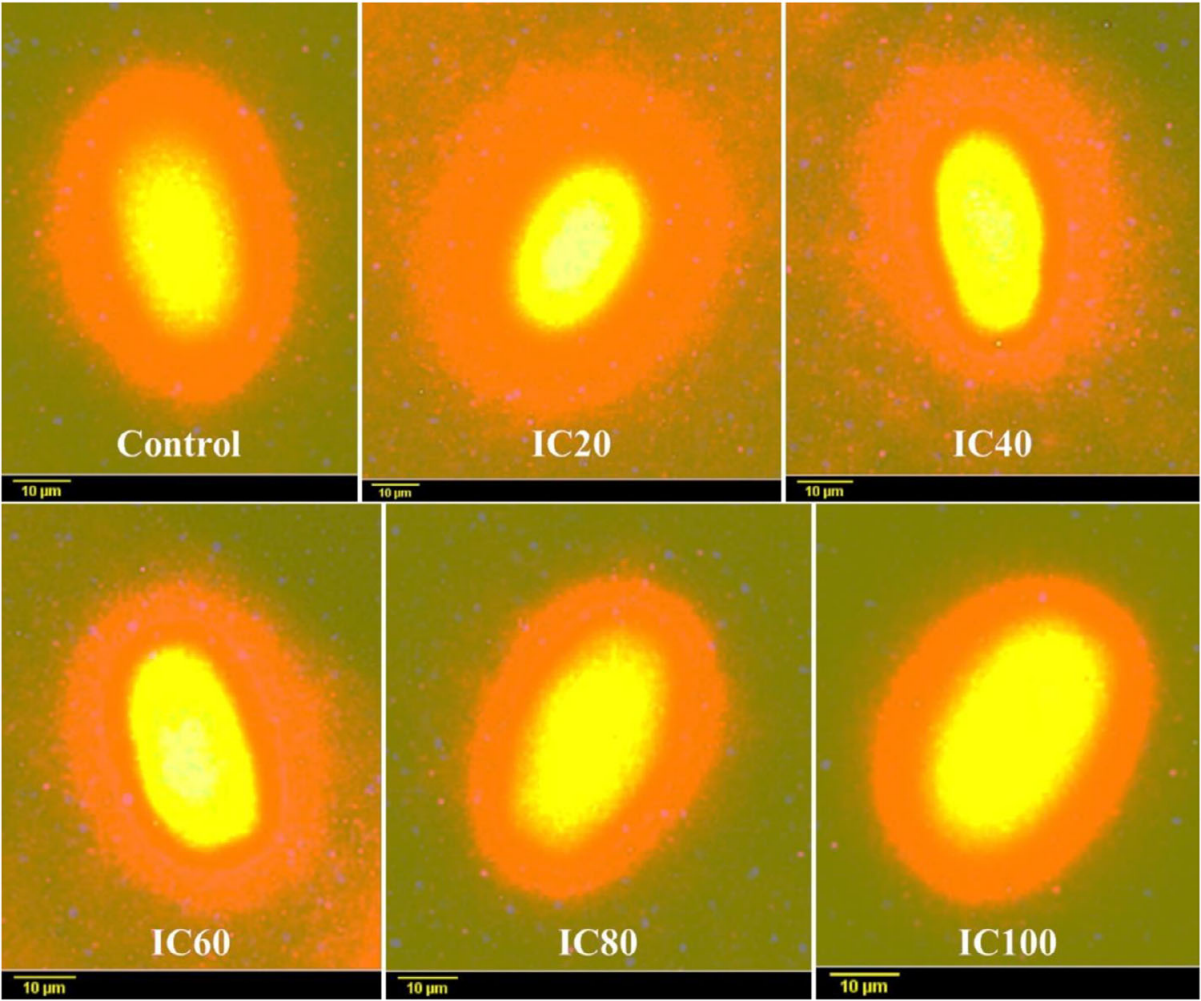
The effects of different concentrations of intracellular compounds of *Lactobacillus plantarum* (ICL) on DNA fragmentation levels in cryopreserved goat sperm.

The results of the SCD parameter are presented in Table 3 and Figure 3. According to these results, ICL20 treatment exhibited a significantly higher halo‐to‐nucleus ratio compared to the control, ICL40, ICL60, ICL80 and ICL100 treatments. In addition, ICL40 treatment showed a significantly higher halo‐to‐nucleus ratio than ICL100 treatment.

## 4 Discussion

In the past decade, probiotic bacteria have gained considerable attention and have emerged as a novel approach for the treatment of various disorders (X. Tian et al. 2019). Scientific evidence indicates that certain LAB can play a significant role in promoting health when administered as probiotics (Murata et al. 2025; Sarita et al. 2025). Moreover, these bacteria have been shown to possess antioxidant properties, effectively reducing oxidative stress (Łepecka and Kołożyn‐Krajewska 2025).

The beneficial effects of LAB are mediated through multiple mechanisms. For instance, bacterial products or cellular components can act as bioactive compounds (Kobatake et al. 2017). In the present study, the ICL were extracted according to previously described methods (Schelli et al. 2017; Xu et al. 2017; Yang et al. 2018; S. H. Kim et al. 2023) and analysed via GC–MS to identify constituent compounds. The analysis revealed that compounds such as 1‐benzenesulfonyl‐1H‐pyrrole and benzenesulfonyl azide were the most abundant among the intracellular components, suggesting that these compounds may exert protective effects on sperm cells and contribute to reduced LPO. The molecular structure of 1‐benzenesulfonyl‐1H‐pyrrole suggests the presence of distinct hydrophobic and hydrophilic moieties, implying potential amphiphilic behaviour. The benzene ring confers hydrophobic character, consistent with prior studies highlighting the lipophilic nature of benzene derivatives and their ability to partition into nonpolar environments (Blank and McAuliffe 1985; Gajjar and Kasting 2014). The sulfonyl group exhibits pronounced polarity and hydrogen‐bonding capability, indicative of hydrophilic interactions (Abhayawardhana et al. 2014). Notably, the pyrrole ring presents a dual character, capable of interacting with both polar and nonpolar phases, potentially acting as a modulating unit that balances the hydrophobic and hydrophilic regions, as reported for pyrrole‐containing compounds and macrocycles (Rusu et al. 2024; Ly et al. 2024), collectively suggesting that 1‐benzenesulfonyl‐1H‐pyrrole may possess amphiphilic properties. Among the intracellular compounds of ICL present at lower relative abundance, 4‐ethyl‐3‐methyl‐9H‐carbazole‐2‐carboxylate warrants consideration. Based on previous reports demonstrating that carbazole‐containing frameworks can be engineered as amphiphilic molecules capable of self‐assembly and direct membrane interaction, this compound may likewise exhibit amphiphilic behaviour, arising from the coexistence of a hydrophobic polyaromatic carbazole core and polar functional moieties within its structure (X. Li et al. 2018; Lin et al. 2020). Amphiphilic compounds, characterized by their dual hydrophilic‐hydrophobic nature, have been shown to interact with lipid bilayers and cellular membranes, potentially stabilizing membrane architecture, preserving fluidity and protecting cells from oxidative damage (Marie et al. 2014). Soy lecithin, a naturally occurring phospholipid mixture, represents a prime example of a biologically relevant amphiphilic compound. Its hydrophilic headgroups engage with the aqueous cytoplasm, whereas its hydrophobic tails integrate into lipid membranes, thereby promoting membrane stability and mitigating LPO. This structural versatility supports cellular homeostasis and resilience under stress conditions (Dalmazzo et al. 2018). Similarly, alpha‐lipoic acid (ALA) exhibits amphiphilic properties due to its solubility in both hydrophilic and lipophilic environments. Owing to its antioxidant properties and amphiphilic nature, ALA can neutralize ROS, regenerate endogenous antioxidants such as glutathione and vitamins C and E and interact with cellular membranes to preserve structural integrity and stability (Shahid et al. 2025). In general, amphiphilic compounds contribute to the protection of membrane architecture, attenuation of oxidative stress and enhancement of cellular resilience under physiological and pathological challenges (Marie et al. 2014). Within this context, 1‐benzenesulfonyl‐1H‐pyrrole, identified as the most abundant component of the ICL compounds, together with the lower‐abundance compound 4‐ethyl‐3‐methyl‐9H‐carbazole‐2‐carboxylate, may exhibit amphiphilic behaviour based on their structural features. Such amphiphilic behaviour could facilitate interactions with membrane lipid components, thereby contributing to membrane stabilization and providing a plausible mechanistic explanation for the observed improvements in sperm quality parameters during the freezing–thawing process. Benzenesulfonyl azide is an aromatic sulfonyl azide with predominantly hydrophobic character, and may contribute to amphiphilic behaviour when combined with suitable hydrophilic components (E. Tian et al. 2022; Luo et al. 2024). In the present formulation, this amphiphilic tendency may potentially act synergistically with other lipophilic and amphiphilic compounds of ICL, as well as with hydrophilic components of the extender, such as glycerol and Tris, or citric acid, and lipophilic constituents like egg yolk phospholipids and lipoproteins. These interactions may facilitate coordinated engagement with both hydrophobic and hydrophilic domains of the sperm membrane, which could enhance membrane stability, limit LPO and contribute to a protective interfacial layer that improves sperm resistance to cold shock and oxidative stress during cryopreservation and thawing. Furthermore, 4′‐methoxyacetophenone oxime, present at low levels in the ICL, has been reported by Barghouthi et al. (2017) to exhibit antibacterial activity at low concentrations, suggesting that its presence may potentially play a role in reducing microbial contamination during sperm cryopreservation and thawing. In addition, cyclohexane, 1‐ethyl‐1‐methyl‐, which is present in low amounts in ICL, also exhibits antimicrobial properties (Harikrishnan et al. 2010). Moreover, in the identified ICL compounds, a pyrrole ring is present within the molecular structure; pyrrole‐containing frameworks are frequently reported in biologically active natural and synthetic molecules, mainly due to their balanced physicochemical properties, which combine moderate lipophilicity with the ability to participate in non‐covalent interactions. This dual character facilitates passive diffusion across biological membranes and promotes molecular compatibility with lipid‐rich environments. In the present ICL profile, pyrrole occurs both as a directly substituted heterocycle, as in 1‐benzenesulfonyl‐1H‐pyrrole, and as an embedded nitrogen‐containing unit within a fused aromatic system, such as 4‐ethyl‐3‐methyl‐9H‐carbazole‐2‐carboxylate. Although these compounds differ structurally, the presence of a pyrrolic nitrogen is a common feature that may contribute to membrane interaction, molecular stabilization and modulation of the intracellular environment. Such properties are consistent with reported roles of pyrrole‐based motifs in enhancing cellular permeability, facilitating biological interactions and indirectly supporting protective mechanisms in complex biological systems. Therefore, within the context of sperm cryopreservation, pyrrole‐containing compounds in the ICL may act as auxiliary molecular components that help maintain membrane integrity and cellular functionality during freezing and thawing (Ivan et al. 2022; Noti et al. 2023; Rusu et al. 2024; Ly et al. 2024). Consistent with our findings, S. H. Kim et al. 2023) reported that *L. plantarum* intracellular extracts contain a diverse array of bioactive compounds. Using multi‐platform mass spectrometry (GC‐TOF‐MS, UHPLC‐LTQ‐Orbitrap‐MS, LC‐triple‐Q‐MS), they identified amphiphilic hydroxy fatty acids (including hydroxy‐[methylthio]butanoic acid and hydroxyisovaleric acid) and pantothenic acid. While the specific compounds do not directly overlap with those in our study, their identification highlights a comparable repertoire of intracellular metabolites that, based on our results, may contribute to cellular protective effects. Based on these results and supporting literature, the positive effects of ICL on goat semen were expected. In our study, low concentrations of ICL (ICL20 and ICL40) significantly improved sperm motility, viability, membrane integrity, MDA levels and DNA integrity. For example, progressive motility increased from 35% in the control group to 44% in the ICL20 treatment. Conversely, higher concentrations (ICL80 and ICL100) exerted adverse effects on sperm quality, likely due to high‐dose toxicity or negative impacts of certain bioactive compounds. Notably, ICL20 and ICL40 treatments outperformed ICL100 in parameters such as membrane integrity, viability, MDA, DNA integrity and several motility indices, highlighting that high doses of ICL can negatively affect sperm quality during cryopreservation. These observations align with the dose–response principle in biology, indicating that low concentrations of bioactive compounds may exert protective effects, whereas higher doses can induce toxicity (Calabrese 2009). Similarly, Marie et al. (2014) reported that at elevated concentrations, amphiphilic macromolecules transition from forming protective layers on cell membranes to disrupting membrane organization and inducing excessive permeabilization, leading to cellular content leakage and cytotoxicity. This membrane destabilization is closely associated with oxidative stress‐related processes, including LPO, and provides a mechanistic basis for the detrimental effects of high ICL doses observed in the present study on sperm membrane integrity, viability and overall quality. Therefore, determining the optimal dose for ICL supplementation in sperm cryopreservation is essential.

This study aimed to investigate the protective effects of ICL on goat sperm during cryopreservation. Among the multiple indices of sperm quality, motility characteristics are critical determinants of fertility (Abou‐El‐Naga et al. 2025). Given that cryopreservation and thawing markedly reduce these features, assessment of motility parameters under such conditions is particularly important (Zang et al. 2025). Our results indicated that the ICL20 treatment significantly increased total sperm motility compared to the control (*p* < 0.05). Furthermore, both ICL20 and ICL40 treatments significantly enhanced progressive motility. ALH was significantly higher in ICL20 and ICL40 than in the control, and ICL20 also significantly increased BCF. Similarly, ICL20 and ICL40 treatments significantly improved sperm viability relative to the control.

LPO can disrupt the lipid matrix of sperm membranes, leading to reduced motility and compromised membrane integrity (Abah et al. 2025). MDA, a primary by‐product of unsaturated fatty acid oxidation, is widely used as an indicator of oxidative damage (Petway et al. 2025; Ezati et al. 2025). Therefore, assessing MDA is critical in studies evaluating sperm quality. Our findings demonstrated that ICL20 and ICL40 treatments significantly reduced MDA concentrations compared to the control. Previous studies have shown that cryopreservation and thawing can alter sperm chromatin structure (Paoli et al. 2019; Tamburrino et al. 2025) and that oxidative stress and free radical production can negatively impact sperm DNA integrity (Humaidan et al. 2022; Henkel 2024; Fleming and Thomson 2025). Considering the vital role of DNA integrity in fertilization success and embryo development, we assessed sperm DNA using the SCD assay. ICL20 exhibited the lowest DNA fragmentation among treatments, indicating significant protective effects. In agreement with our results, Aguilar‐Toalá et al. (2019) demonstrated that intracellular components of *Lactobacillus* reduce oxidative stress‐induced haemolysis in erythrocytes by preserving membrane integrity and limiting LPO. Given the structural and functional vulnerability of sperm membranes to LPO during cryopreservation, these findings further confirm that ICL helps stabilize sperm membranes and exerts protective effects against oxidative damage during cryopreservation. These results are consistent with Dim et al. (2020), who reported that probiotic supplementation improved semen volume, sperm concentration, progressive and total motility and the proportion of live and morphologically normal sperm in turkeys.

Additional studies support the protective effects of *L. plantarum*. Oral administration of *L. plantarum* mitigated testicular damage caused by diethyl hexyl phthalate (DEHP) in male mice, improving serum testosterone, semen quality and sexual organ development (X. Tian et al. 2019). *L. plantarum*, alone or with inulin, enhanced sperm motility and viability in diabetic male mice by promoting Leydig cell proliferation, spermatid counts and seminiferous tubule growth (Rahimiyan‐Heravan et al. 2020). *Lactobacillus casei* treatment reduced MDA levels in serum and liver while increasing antioxidant enzymes (SOD and GSH‐Px) in hyperlipidaemic rats (Y. Zhang et al. 2010). Furthermore, *Lactobacillus* metabolites protect human sperm from iron‐induced LPO, preserving motility and viability under oxidative stress and shielding sperm from ROS (Barbonetti et al. 2011).

However, some studies report inconsistent results. Probiotic supplementation in pigs with colitis did not improve protein synthesis, oxidative stress or antioxidant capacity (Harding et al. 2008). Nevertheless, the intracellular metabolites of ICL exhibit potent protective effects, interacting with sperm membranes to reduce oxidative damage and limit LPO during cryopreservation. According to Aguilar‐Toalá et al. (2019), these compounds act as lipophilic antioxidants, preventing membrane damage via hydrogen atom transfer or single‐electron transfer to proteins and lipids, which is consistent with the protective effects of ICL on sperm membrane integrity and reduction of oxidative damage observed in the present study.

Moreover, other studies have also reported that these metabolites exhibit antioxidant and radical‐scavenging activities, supporting their beneficial effects.

For example, *L. plantarum* isolated from fermented Japanese radish exhibited fivefold higher superoxide anion scavenging capacity than unfermented radish juice (Kuda et al. 2010). A fermented solution containing *L. brevis* and seaweed displayed strong antioxidant activity in DPPH, superoxide and xanthine oxidase assays (B. J. Lee et al. 2010). *L. plantarum* from Narezushi fermentation‐enhanced antioxidant capacity in milk, soy milk and vegetables (Kanno et al. 2012). *L. plantarum* supplementation in aged mice under oxidative stress increased serum SOD, GSH‐Px and total antioxidant capacity and reduced MDA levels (S. Li et al. 2012). *L. plantarum* DM5 isolated from traditional fermented beverages effectively scavenged DPPH, superoxide and hydroxyl radicals while preventing ascorbate oxidation (Das and Goyal 2015). These findings collectively indicate that ICL can exert protective effects against ROS‐induced oxidative damage and contribute to the reduction of LPO.

Moreover, some *Lactobacillus* species can adsorb and remove heavy metals such as cadmium, lead and copper under laboratory conditions (Halttunen et al. 2008; Mrvcic et al. 2009). The cytoplasmic fraction of *L. plantarum* possesses anti‐tumour properties in mice (J. Y. Kim et al. 2002). These protective properties are particularly relevant for safeguarding sperm cells from oxidative and heavy‐metal‐induced stress during cryopreservation. Overall, our findings indicate that the ICL act as a natural protector against environmental and oxidative stress, improving sperm quality, especially under challenging conditions such as freezing.

Despite the promising protective effects of ICL on goat sperm during cryopreservation, this study has limitations. Notably, fertility outcomes—such as artificial insemination or in vitro fertilization—could not be evaluated due to the lack of specialized laboratory equipment. While post‐treatment sperm quality was comprehensively assessed in terms of motility, viability, membrane and acrosome integrity, LPO (MDA level) and DNA damage, the direct impact of ICL on fertilization potential remains unclear. Future studies should include fertility assessments to fully confirm the protective effects of ICL.

## 5 Conclusions

The results of the present study demonstrated that ICL can serve as an effective protective agent in goat semen extender, enhancing sperm quality during cryopreservation and thawing. Application of appropriate concentrations, particularly ICL20 and ICL40, improved sperm motility, viability, membrane integrity and DNA integrity, and also reduced LPO (MDA levels). These beneficial effects are likely associated with the antioxidant and protective properties of ICL, which help mitigate oxidative stress, neutralize free radicals and reduce LPO. In contrast, higher concentrations of ICL (ICL80 and ICL100) exerted detrimental effects on sperm quality, highlighting the importance of optimizing the dosage. These findings are consistent with previous studies and suggest that ICL can serve as a natural and safe additive to improve sperm cryopreservation. Nevertheless, further research is required to elucidate the precise mechanisms underlying these effects and to assess their long‐term outcomes.

## Author Contributions

Farshad Ariyan, Amjad Farzinpour, and Abbas Farshad contributed substantially to the conception and design of the study. Data acquisition was performed by Farshad Ariyan and Aram Sharifi. Data analysis and interpretation were carried out by Farshad Ariyan, Amjad Farzinpour, Abbas Farshad, and Aram Sharifi. The initial draft of the manuscript was prepared by Farshad Ariyan, and critical revision for important intellectual content was conducted by Amjad Farzinpour. All authors have reviewed and approved the final version of the manuscript for publication.

## Funding

The authors have nothing to report.

## Ethics Statement

All experimental procedures in this study were conducted in accordance with international guidelines and were approved by the Animal Care and Use Committee of the University of Kurdistan, Sanandaj, Kurdistan, Iran (Ethical Approval Code: **IR.UOK.REC.1404.021**).

## Conflicts of Interest

The authors declare no conflicts of interest.

## Data Availability

Data supporting the findings of this study are available from the corresponding author upon reasonable request.
